# Consuming Genistein Improves Survival Rates in the Absence of Laxative in ΔF508-CF Female Mice

**DOI:** 10.3390/nu10101418

**Published:** 2018-10-03

**Authors:** Ryan Lord, Nathan Fairbourn, Charisma Mylavarapu, Ammer Dbeis, Taylor Bowman, Archana Chandrashekar, Tatum Banayat, Craig A. Hodges, Layla Al-Nakkash

**Affiliations:** 1 Department of Physiology, AZCOM, Midwestern University, 19555 N. 59th Avenue, Glendale, AZ 85308, USA; rlord98@midwestern.edu (R.L.); nfairbourn48@midwestern.edu (N.F.); cmylavarapu15@midwestern.edu (C.M.); adbeis39@midwestern.edu (A.D.); tbowman93@midwestern.edu (T.B.); achandrashekar85@midwestern.edu (A.C.); tbanay@midwestern.edu (T.B.); 2 Department of Genetics & Genome Sciences & Department of Pediatrics, Case Western Reserve University, 10900 Euclid Avenue, 830 BRB, Cleveland, OH 44106, USA; craig.hodges@case.edu

**Keywords:** genistein, ∆F508-CF, mouse, survival

## Abstract

Genistein is a naturally occurring isoflavone found in soy. Genistein has been shown to increase the open probability of the most common cystic fibrosis (CF) disease-associated mutation, ∆F508-CFTR. Mice homozygous for the ∆F508 mutation are characterized with severe intestinal disease and require constant laxative treatment for survival. This pathology mimics the intestinal obstruction (meconium ileus) seen in some cystic fibrosis patients. This study tested whether dietary supplementation with genistein would reduce the dependence of the ∆F508 CF mouse model on laxatives for survival, thereby improving mortality rates. At weaning (21 days), homozygous ∆F508 mice were maintained on one of three diet regimens for a period of up to 65 days: normal diet, normal diet plus colyte, or genistein diet. Survival rates for males were as follows: standard diet (38%, *n* = 21), standard diet plus colyte (83%, *n* = 42) and genistein diet (60%, *n* = 15). Survival rates for females were as follows: standard diet (47%, *n* = 19), standard diet plus colyte (71%, *n* = 38), and genistein diet (87%, *n* = 15). Average weight of male mice fed genistein diet increased by ~2.5 g more (*p* = 0.006) compared to those with colyte treatment. Genistein diet did not change final body weight of females. Expression of intestinal SGLT-1 increased 2-fold (*p* = 0.0005) with genistein diet in females (no change in males, *p* = 0.722). Expression of GLUT2 and GLUT5 was comparable between all diet groups. Genistein diet reduced the number of goblet cells per micrometer of crypt depth in female (*p* = 0.0483), yet was without effect in males (*p* = 0.7267). The results from this study demonstrate that supplementation of diet with genistein for ~45 days increases the survival rate of female ∆F508-CF mice (precluding the requirement for laxatives), and genistein only improves weight gain in males.

## 1. Introduction

Cystic fibrosis (CF), the most common recessive lethal genetic disorder in Caucasians is a result of mutations in the cystic fibrosis transmembrane conductance regulator (CFTR) gene [1,2,3,4]. The consequences of these mutations are multifarious due to the defective function of the ubiquitous CFTR protein: chronic respiratory tract infections, pancreatic exocrine insufficiency and intestinal obstruction [3]. Clinically, this manifests as meconium ileus in some CF newborns and distal intestinal obstructive syndrome (DIOS) in older CF patients [5,6]. The CF mouse demonstrates intestinal complications comparable to those seen in CF patients; intestinal obstruction attributed to formation of mucus plugs, along with dysfunctional absorptive and secretory functions [7,8,9], which results in increased morbidity of the mice [10,11]. The intestinal impactions observed in the CF (ΔF508 homozygous) mouse necessitate a constant laxative treatment (colyte) for survival [9,12,13].

The search for pharmacological agents to promote CFTR activity and thus improve CF tissue function and overall CF health led to this study, which aimed to investigate the role that genistein (a naturally occurring isoflavonic phytoestrogen found in high concentrations in soy products, legumes and grains) could play [14,15,16]. Epidemiologic studies have suggested multiple health benefits for humans consuming soy-based foods, including reduced risk of cancers [17,18], reduced incidence of coronary artery disease [19,20], reduced cholesterol levels [21] and reduced blood pressure [22]. The cellular targets and mechanisms underlying the multiple health benefits of soy products are relatively unclear in large part due to its tissue-specific and sex-specific effects. 

Genistein is structurally similar to estrogen, and a recent review provides structural comparisons of several natural compounds along with support for the use of genistein as a therapeutic agent in the treatment of CF [23]. In addition, genistein has been shown to increase the open probability (Po) of the most common cystic fibrosis disease-associated mutation, ∆F508-CFTR, to levels comparable to those seen in wild-type, Wt-CFTR [24,25,26]. Recently, demonstration of partial rescue of S1045Y-CFTR surface expression and function by genistein prompted the proposed use of genistein to ameliorate CF-related symptoms in individuals with the S1045Y-CFTR mutation [27]. Moreover, recent studies using rectal organoids provide promising support for genistein used in combination with curcumin and VX-770, with the CF-associated mutations S1251N, ΔF508 and G551D [28]. These data suggest that genistein may have therapeutic potential for treatment of CF. Moreover, previous studies have shown that genistein can improve ∆F508-CFTR in in vitro cell systems with an EC50 of 5 µM [29,30] which is well within the physiological range attainable by dietary modifications [31]. Genistein is readily absorbed across the intestines and can readily reach micromolar concentrations in the serum [32]. Indeed, mice consuming 750 ppm genistein generate plasma genistein concentrations of ~2 µM [33]. Previously, the effect of chronic dietary genistein (for 4 weeks) on intestinal function in wild-type mice was examined [34], and it was demonstrated that consumption of 600 mg genistein/kg diet increased serum levels of genistein comparably in wild-type males and females (~5 µM), resulting in a significant increase in basal jejunum transepithelial short-circuit current (a measure of chloride secretion) [34]. These serum levels are comparable to those obtained with a soy milk diet in humans, which also results in plasma genistein concentrations of ~2 µM [31,35].

The aim of the study was to examine whether or not chronic exposure to elevated levels of dietary genistein, for a period of 4 weeks, would increase survival of the CF mouse.

## 2. Materials and Methods

CF mouse model: The ΔF508 (CF) mice used in this study were generated by targeted replacement of the wild-type exon 10 allele with the ΔF508 mutant allele. This manipulation also resulted in the neomycin phosphotransferase gene inserted in intron 10 as previously described [36]. Mice were genotyped from tail clip DNA, using primers and methods previously described in detail [36,37]. CF mice were randomly assigned to one of three diet groups—600 mg genistein/kg diet (*n* = 15 for males and females each) or standard (normal) diet either with colyte (*n* = 42 males, *n* = 38 females), or without colyte (*n* = 21 males, *n* = 19 females)—and maintained on one of these three diets until day 65 or until demise. Colyte is polyethylene glycol 3350 with electrolytes (Kremers Urban, Princeton, NJ, USA). Colyte is an electrolyte solution commonly used as a laxative for CF mice [12] and clinically in the management of constipation [38]. Specialized genistein-containing diet was prepared by Dyets Inc. (Bethlehem, PA, USA) [34]. At euthanasia, the tissues from these original mice used in the survival studies (*n* = 15–42/group) were collected (i.e., jejunum) and subsets of these groups (between 4–8/group) used for determinations of western blot and histology. 

Mice were housed individually, in an animal care facility with 12:12-h light-dark cycle and fed/watered ad libitum until day 65. Body weight was measured regularly (every 5 days) during the diet study and general health monitored biweekly. Body weight was measured once by placing each individual mouse in a small container on a balance (pre-zeroed) and waiting for the mouse to be calm, to enable steady readings. If excessive movement of the mouse was noted, it was taken out and reweighed. The balance was regularly checked for accuracy (using a 5 g weight) throughout the study, specifically on those dates that the mouse weights were taken. CF mice were cared for in accordance with Case Western Reserve University (CWRU) Institutional IACUC, guidelines. IACUC title: breeding mouse models of cystic fibrosis, protocol #: 2014-0064, approval date April 2017, CWRU. This animal care protocol has an IACUC-approved amendment to include the use of genistein for this study. The authors ensure that this research complies with the commonly accepted practices of replacement of animals by alternatives where possible, reduction in numbers of animals used, and refinement of experimental conditions and procedures to minimize harm to animals. The authors adhered to the guidelines set forth in Animals (Scientific Procedures) Act 1986: Code of practice for the housing and care of animals used in scientific procedures [39]. 

### 2.1. Histology 

Freshly isolated segments of jejunum were fixed in sucrose overnight and then embedded in paraffin. Sections (8 µM) were stained for mucin, with Alcian Blue/Periodic Acid Schiff (PAS) using standard methods. Jejunum morphology (villi length, numbers of goblet cells/villi, crypt depth and number of goblet cells/crypt) measurements were made using Axiovision (Carl Zeiss). To normalize the morphology data, comparisons were made of numbers of goblet cells/µm crypt length and of the numbers of goblet cells/µm villi length. Averages of measurements were taken from 10 separate images of jejunum/mouse, and data are presented as the average of multiple mice (*n* = 6) in each diet group. 

### 2.2. Western Blot Analysis 

At collection, jejuna were immediately snap frozen in liquid nitrogen and stored at −80 °C. Jejuna were later prepared for western blot analysis by homogenization, and the western blot protocol used was similar to that described previously [40,41,42]. Blots were incubated with primary antibody to facilitated glucose transporter solute carrier-2, GLUT2 (1:500, ~60 kDa, Santa Cruz, CA, USA), facilitated glucose transporter solute carrier-5, GLUT5 (1:200, ~45–60 kDa, Santa Cruz, CA, USA), or sodium-dependent glucose cotransporter-1, SGLT-1 (1:200, ~61 kDa, Cell Signaling, Danvers, MA, USA) overnight at 4 °C. After washing, blots were incubated with secondary antibody, anti-rabbit IgG (H + L) Dylight (1:15,000, Thermo Scientific, Rockford, IL, USA), for 1 h at room temperature. To re-probe for actin, blots were incubated with anti-GAPDH primary antibody (1:4000, ~40 kDa, Thermo Scientific, Rockford, IL, USA) for 1 h at room temperature. Blots were washed and then re-incubated with the appropriate secondary antibody anti-mouse IgG (H + L) (1:15,000, Dylight, Thermo Scientific Rockford, IL, USA). Images of membranes were taken with all proteins of interest normalized to GAPDH. Band density was analyzed (from *n* = 4–8 mouse samples/per diet group, and each mouse band density/protein of interest is used once) using Odyssey-Clx (LI-COR, Lincoln, NE, USA) and Image Studio (LI-COR, Lincoln, NE, USA).

### 2.3. Statistics 

Data are expressed as mean ± standard deviation (SD). Numbers in parentheses are numbers of tissues used from separate individual mice. Kaplan-Meier survival curves were evaluated using a log-rank test. Data were checked for normality and distribution was found to be normal. Comparisons between diet groups were performed using one-way ANOVA with post-hoc Neuman-Keuls test (for a normal distribution of data) using GraphPad (GraphPad Software, Inc., La Jolla, CA, USA). 

## 3. Results

### 3.1. Physical Characteristics of the Mice

**Figure 1:**
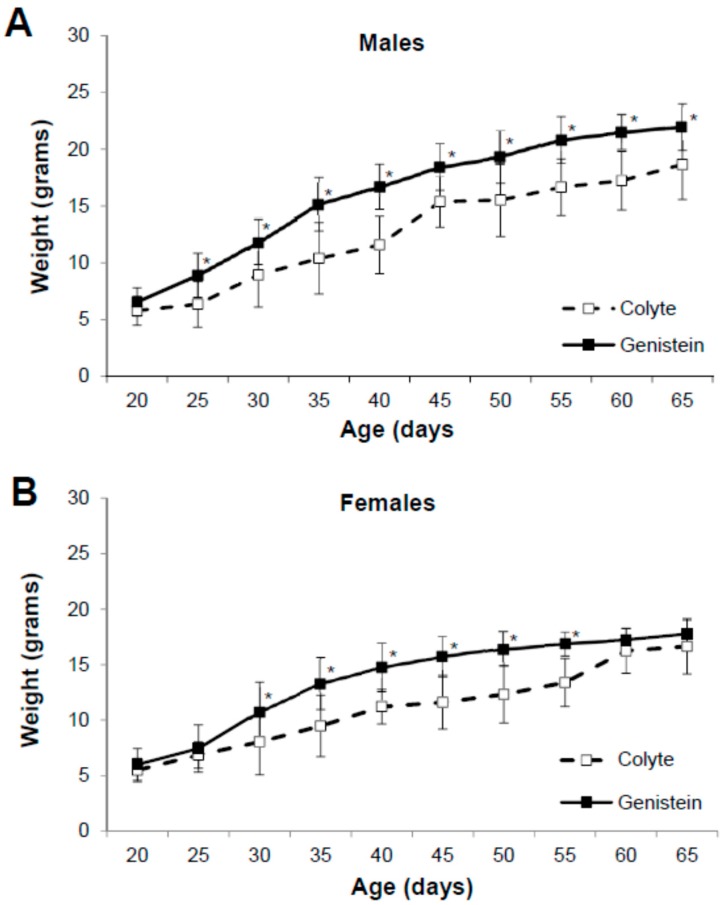
Effect of dietary genistein on body weight of ΔF508-CF mice. Body weight was measured weekly from weaning (aged 20 days) and monitored up to age 65 days. (**A**) Weight of males on colyte (*n* = 8) and genistein diet (*n* = 14). (**B**) Weight of females on colyte (*n* = 9) and genistein diet (*n* = 15). Data are mean ±SD. * indicates significant genistein-mediated difference, *p* < 0.05.

The effect of ad libitum feeding of the genistein-containing diet, the standard diet with colyte, or the standard diet alone on the growth of ΔF508 male and female mice was ascertained. Mice entered the diet study at weaning (day 20) and were randomly separated into one of the three diet groups. During the diet study (from day 20 to 65), all mice maintained a steady increase in weight gain (Figure 1). Average weight of male mice on colyte increased from 5.79 ± 1.27 g to 18.66 ± 3.07 g (*n* = 8), and the average weight of male mice fed genistein diet increased from 6.55 ± 1.25 g to 21.96 ± 2.05 g (*n* = 14). Genistein diet significantly increased both rate of weight-gain and final body weight compared to colyte-treated (age 25 days, *p* = 0.0087; 30 days, *p* = 0.02; 35 days, *p* = 0.0028; 40 days, *p* = 0.0003; 45 days, *p* = 0.0036; 50 days, *p* = 0.0067; 55 days, *p* = 0.0003; 60 days, *p* = 0.0002; 65 days, *p* = 0.006, Figure 1A). Average weight of female mice on colyte increased from 5.50 ± 1.05 g to 16.65 ± 2.49 g (*n* = 9), and the average weight of female mice fed genistein diet increased from 6.01 ± 1.43 g to 17.77 ± 1.24 g (*n* = 15). While average weight of females on genistein was significantly greater than females on colyte from 30–55 days of age (30 days, *p* = 0.03; 35 days, *p* = 0.0022; 40 days, *p* = 0.0025; 45 days, *p* = 0.0001; 50 days, *p* = 0.0001; 55 days, *p* = 0.0001), genistein diet did not change the final body weight of females (Figure 1B).

**Figure 2:**
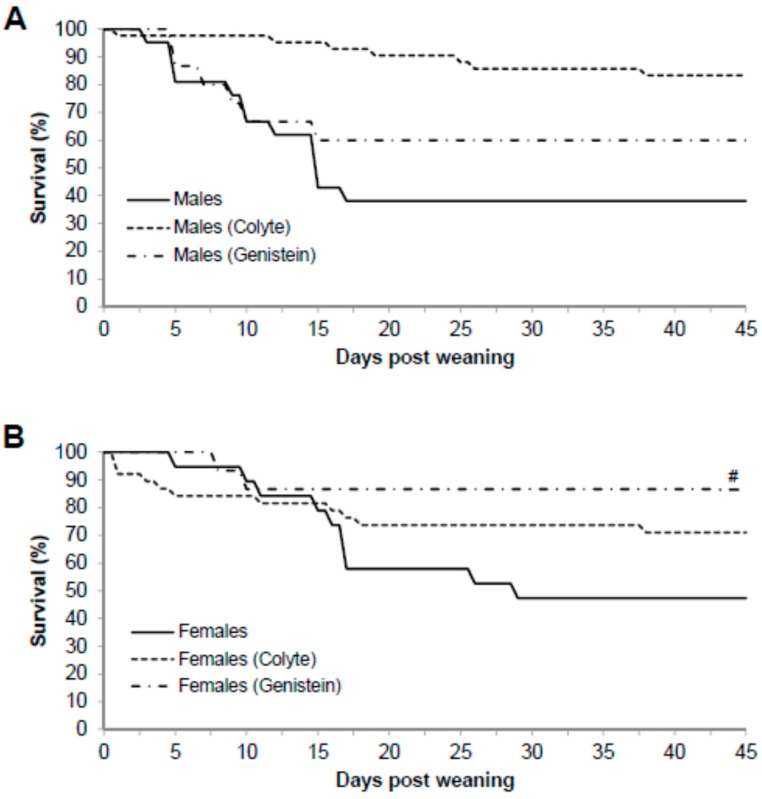
Effect of dietary genistein on survival rates of ΔF508-CF mice. Differing diets were started at weaning (aged 20 days), monitored up to age 65 days. (**A**) The percentage of males surviving with each diet were; standard diet (38%), colyte diet (83%) and genistein diet (60%). (**B**) The percentage of females surviving with each diet were; standard diet (47%), colyte diet (71%), and genistein diet (87%). Data are mean. # indicates significant genistein-mediated difference compared to controls, *p* < 0.05.

Mice homozygous for the ΔF508 mutation (CF mice) are characterized by severe intestinal disease and therefore require constant laxative treatment for survival. This study therefore tested whether dietary genistein would reduce the dependence of the ΔF508 CF mouse model for laxative treatment for survival. Survival rates for males were as follows: fed standard diet (38%, 8/21), fed standard diet plus colyte (83%, 35/42), and fed genistein diet (60%, 9/15). Thus, for male ΔF508-CF mice, genistein diet did not improve survival rate compared to those on colyte (*p* = 0.0163) and indeed significantly worsened survival rate with genistein compared to colyte. Genistein did not improve survival rate in males compared to those fed standard diet (*p* = 0.38, Figure 2A). Survival rates for females were as follows: fed standard diet (47%, 9/19), fed standard diet plus colyte (71%, 27/38), or fed genistein diet (87%, 13/15). Thus, survival rate of female ΔF508 mice fed genistein diet was significantly higher when compared to those fed standard diet (*p* = 0.005), but not those fed colyte (*p* = 0.07, Figure 2B).

### 3.2. Jejunum Morphology

**Figure 3:**
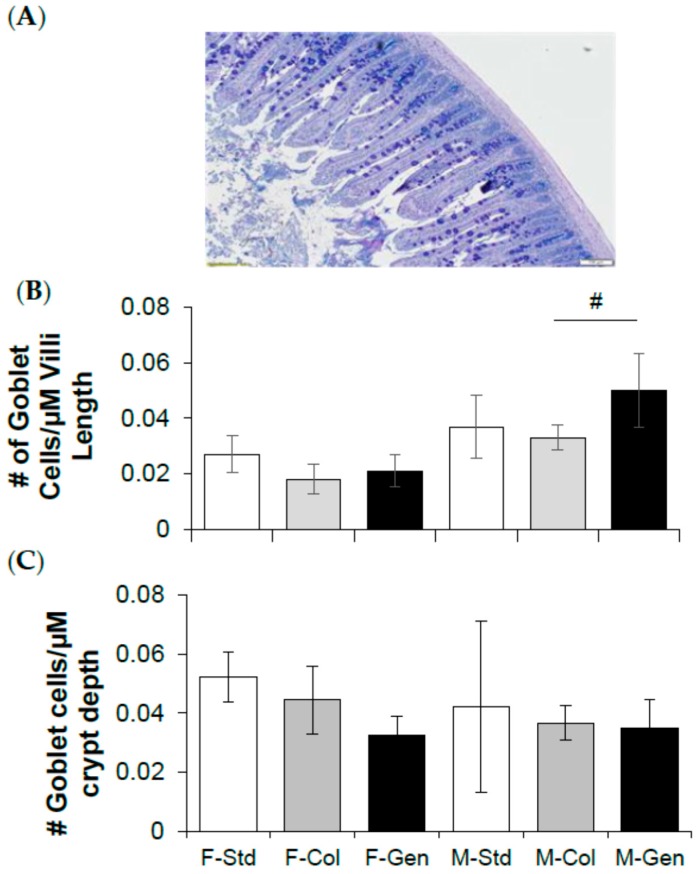
Effect of dietary genistein on jejunum morphology of ΔF508-CF mice. (**A**) Representative alcian blue stained section from jejunum. (**B**) Average number of goblet cells per micrometer of villi length (*n* = 6/group). (**C**) Average number of goblet cells per micrometer of crypt depth (*n* = 6/group). Note: Std = standard diet, Col = colyte treated, and Gen = genistein diet. Data are mean ± SD. ^#^ indicates significant genistein-mediated difference compared to colyte-treated, *p* < 0.05.

In theory, modifications in goblet cell numbers and presumably mucin production could have effects on intestinal luminal fluidity and impaction. Therefore, the current study tested the prediction that genistein diet could have beneficial effects, i.e., reduce goblet cell number, which could contribute towards the lack of impaction of genistein fed mice. Histological sections were stained using Alcian Blue/Periodic Acid Schiff and analyzed for crypt depth and villi length and the numbers of goblet cells. There was no change in villi length among the female groups (F-Std = 339.10 ± 66.19 µm, F-Col = 382.70 ± 90.28 µm, F-Gen = 405.93 ± 46.98 µm, *P* = 0.3626, n = 6/group), and no change between the male groups (M-Std = 314.50 ± 21.90 µm, M-Col = 380.26 ± 51.62 µm, M-Gen = 347.73 ± 45.92 µm, *p* = 0.2756, *n* = 6/group). The total number of goblet cells per villus was comparable between the female groups (F-Std = 8.83 ± 3.22, F-Col = 6.48 ± 2.67, F-Gen = 8.19 ± 1.81, *p* = 0.1992, *n* = 6/group). In males, the total number of goblet cells was significantly higher 1.37-fold (*p* = 0.0157) with genistein (M-Std = 10.83 ± 2.55, M-Col = 11.63 ± 1.48, M-Gen = 15.98 ± 3.35, *n* = 6/group). Expressing the number of goblet cells/µm of villus length is shown in Figure 3B. The number of goblet cells per µm villus length was comparable between the female groups (*p* = 0.3286) and in males, the total number of goblet cells/µm villus length was significantly higher 1.5-fold (*p* = 0.0147) with genistein.

Crypt depth in females was significantly higher with genistein diet, 1.28-fold (*p* = 0.0276) in (F-Std = 101.03 ± 11.28 µm, F-Col = 106.50 ± 14.00 µm, F-Gen = 135.85 ± 24.15 µm, *n* = 6/group), and no change between the male groups (M-Std = 129.86 ± 30.28 µm, M-Col = 98.46 ± 13.09 µm, M-Gen = 138.39 ± 54.05 µm, *p* = 0.1040, *n* = 6/group). The total number of goblet cells per crypt was comparable between the female groups (F-Std = 4.97 ± 0.27, F-Col = 4.68 ± 1.14, F-Gen = 4.47 ± 1.51, *p* = 0.5689, *n* = 6/group), and between the male groups (M-Std = 5.07 ± 3.61, M-Col = 3.50 ± 0.52, M-Gen = 4.22 ± 0.95, *p* = 0.3188, *n* = 6/group). Expressing the number of goblet cells/µm of crypt depth is shown in Figure 3C. In females, there were 27% less goblet cells per µm crypt length in the genistein-fed group (*p* = 0.0483), whereas no difference was noted between the male groups (*p* = 0.6461). 

### 3.3. Expression of Key Proteins Involved in Absorption across Jejunum 

**Figure 4:**
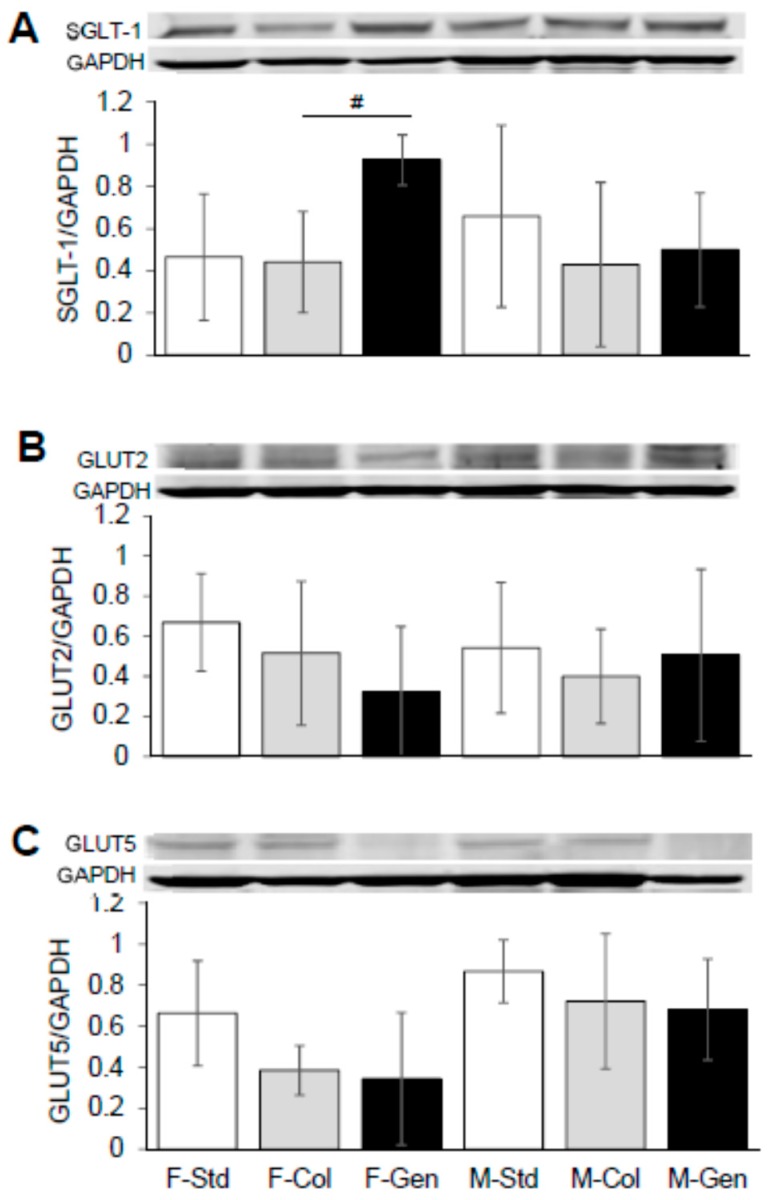
Effect of dietary genistein on total expression of SGLT-1, GLUT2 and GLUT5 in jejunum of ΔF508-CF mice. (**A**) Typical western blot demonstrating SGLT-1 expression (normalized to GAPDH) in jejunum from CF mice. Average SGLT-1/GAPDH ratio is shown comparing regular standard diet, standard diet plus colyte and genistein diet. *n* = 4–7/group. (**B**) Typical western blot demonstrating GLUT2 expression (normalized to GAPDH) in jejunum from CF mice. Average GLUT2/GAPDH ratio is shown comparing regular standard diet, standard diet plus colyte and genistein diet. *n* = 4–8/group. (**C**) Typical western blot demonstrating GLUT5 expression (normalized to GAPDH) in jejunum from CF mice. Average GLUT5/GAPDH ratio is shown comparing regular standard diet, standard diet plus colyte and genistein diet. *n* = 4–8/group. Note: Std = standard diet, Col = colyte treated, and Gen = genistein diet. Values are means ± SD. ^#^ denotes statistical genistein-mediated effect, *p* < 0.05.

Small intestinal absorption across the villi intestinal epithelial membrane requires the activity of the following: sodium-coupled glucose and galactose transport (mediated by SGLT-1), facilitated fructose transport (mediated by GLUT5) across the luminal membrane, along with the facilitated transport of all monosaccharides across the basolateral membrane (mediated via GLUT2). Interestingly, it was shown that SGLT-1 expression was significantly 2-fold higher in mice fed genistein (*n* = 8, *p* = 0.0005), in female mice, compared to those on colyte (*n* = 5), but was without effect in males (*n* = 6, *p* = 0.3900, Figure 4A). These results demonstrate that total protein expression of GLUT5 (for females fed genistein (*n* = 8, *p* = 0.7945) compared to colyte (*n* = 6), and for males fed genistein (*n* = 6, *p* = 1.0) compared to colyte, *n* = 7) and GLUT2 (for females fed genistein (*n* = 8, *p* = 0.4496) compared to colyte (*n* = 7), and for males fed genistein (*n* = 6, *p* = 0.4176) compared to colyte, *n* = 7) was unchanged by genistein-diet for both male and female CF mice (Figure 4B,C). 

## 4. Discussion

This study provides the first evidence that chronic dietary consumption of genistein (600 mg genistein/kg diet), for a period of 4 weeks, increases survival in female ΔF508-CF mice, eliminating the requirement for additional supplemental laxative treatment, and increases body weight of male ΔF508-CF mice. This study concludes that the increased survival rates of female ΔF508-CF mice following 4 weeks on a genistein-rich diet is attributed to at least the following mechanisms: (1) significantly less goblet cells/crypt depth within jejunum, and (2) significantly higher levels of jejunum SGLT-1 expression. 

Genistein has been shown to exhibit beneficial effects on key transporters involved in intestinal epithelial ion transport. For example, acute application of genistein stimulated murine duodenal bicarbonate secretion via a PI3K-depdent pathway [43], and activated a sustained chloride secretion across murine jejunum via a tyrosine-dependent phosphorylation pathway [44]. An examination of genistein’s role on intestinal pacemaker activity reveals contradictory effects; Shin et al. [45] demonstrated that genistein had no effect on normal pacemaker activity in small intestinal interstitial cells of Cajal, yet genistein was shown to reduce colonic pacemaker activity by suppressing calcium oscillations [46], suggesting that genistein’s effects are likely tissue specific. Interestingly, genistein has been shown to improve ΔF508-CFTR channel activity in oocytes and non-CF rectal biopsies; however, it did not induce improvements in chloride secretion across CF airway and intestinal tissues [47]. CFTR potentiators, such as genistein, show promise in clinical treatment regimens for an additional CF-associated mutation A561E [48]. 

Previous studies have suggested that genistein concentrations in plasma with soy-rich diets can reach micromolar levels [49]. Serum concentrations of genistein in the low micromolar range have been obtained in rats and mice after consumption of genistein-containing diets [50,51] resulting in functional changes in tissues: improved basal transepithelial chloride secretion across freshly excised jejunum in wild-type female mice [34] and improved basal transepithelial chloride secretion across freshly excised jejunum from *ob*/*ob* female and male mice [41]. 

For increased survivability, ΔF508-CF mice are routinely placed on laxatives [12]. In the current study, survivability for male and female ΔF508-CF mice over a period of 45 days post weaning, fed normal standard diet without laxative treatment, resulted in low survivability rates (Figure 2). As expected, when subgroups of mice were provided normal standard diet with addition of colyte to drinking water, survivability was greater in males (2.18-fold compared to without colyte) and in females (1.51-fold compared to without colyte). Consuming a genistein-containing diet (in the absence of colyte) in male CF mice was not as beneficial as colyte. On the other hand, female mice fed a genistein diet surpassed their male counterparts’ survivability rates, and even that of either sex on a laxative diet, with a 1.85-fold greater survivability compared to without colyte. Thus, genistein diet generates sex-dependent improvements in survival rates of females compared to males. 

This study provides several notable pieces of evidence supporting the beneficial role of genistein diet on ΔF508-CF mice. (1) Increased weight in CF male mice: It is interesting that consumption of the genistein-diet results in a significantly greater increase in weight gain for the males. While genistein diet increased weight gain at earlier time points in female mice, this treatment diet had no effect on the final weight gain in females. The authors acknowledge that increased weight gain in males is in the absence of positive effects on survival rate. It is of note that there is a clear dearth of published studies examining the role of dietary genistein on murine growth patterns, indeed, genistein (administered for 3 weeks) has been shown to have no effect on growth rates nor on organ morphology in wild-type male and female mice [51]. Discrepancies in that study versus the current study could be related to: murine model (wild-type versus ΔF508-CF mice), duration of exposure (3 versus 4 weeks) or dose of genistein administered (1 g/kg diet versus 600 mg/kg diet). Genistein-mediated effects on muscle weight have previously been reported in male mice; Hirasaka et al. [52] demonstrated that soy isoflavones (genistein and daidzein) significantly increased weight of gastrocnemius muscle in mice (mediated via inhibition of the ERK signaling pathway), after a period of 3 weeks. Thus, it is possible that the CF mice in this study, gained muscle mass resulting in increased total body weight and future studies will address this. (2) Greater SGLT-1 expression in CF female mice: Genistein’s remarkable effect on SGLT-1 expression in females is perhaps not surprising; regulation of SGLT-1 expression by protein kinase A (PKA) and protein kinase C (PKC) has been demonstrated in intestinal tissue [53] and Epidermal Growth Factor has been shown to have stimulatory effects on PKC-regulated glucose absorption [54]. Moreover, genistein treatment can activate the PKC pathway [55]. Whether or not genistein stimulates an increase in expression of SGLT-1 via a PKC-mediated mechanism in female CF mice remains to be elucidated. (3) Fewer goblet cell numbers in CF female mice: less goblet cells and thus a reduction in mucus production would feasibly contribute toward the lack of impaction and increased survivability of the females fed genistein diet. Exposure of mouse epithelial cells to genistein has been shown to significantly reduce Muc-1 expression [56], and whether or not the loss of goblet cell numbers in the current study translates to a reduction in Muc-1 remains to be seen. Previous evidence in broiler chickens have illustrated a dietary genistein-mediated effect on intestinal morphology resulting in increased villus length and crypt depth [57]. Our data therefore indicate species-dependent differences in genistein’s effects on intestinal morphology. A key limitation to the current study is the duration of exposure to dietary genistein (up to 65 days). It is possible that extending the duration of genistein diet for four more weeks, to ~90 days, may have generated varied sex-dependent outcomes in weight gain and survival rates. Future studies will examine this possibility. 

Interestingly, sex-based differences have been documented in patients with CF. Females with CF have a significantly higher mortality than males, resulting in a ~4 year difference in the median age of survival [58,59]. While the reason for this disparity in survival age is presently unknown, our studies indicating a genistein-mediated increase in survival have relevance to the CF female clinical population. 

## 5. Conclusions

In conclusion, this study provides the first evidence that increased consumption of dietary genistein in the absence of laxatives increases survival of female ΔF508-CF mice and increases weight gain in male ΔF508-CF mice. More interestingly, and of particular clinical relevance, it is concluded that these studies will provide a basis for the potential adjunct therapeutic use of dietary genistein in CF.
